# Anti-Hepatocellular Carcinoma (HepG2) Activities of Monoterpene Hydroxy Lactones Isolated from the Marine Microalga *Tisochrysis Lutea*

**DOI:** 10.3390/md18110567

**Published:** 2020-11-19

**Authors:** Katkam N. Gangadhar, Maria João Rodrigues, Hugo Pereira, Helena Gaspar, F. Xavier Malcata, Luísa Barreira, João Varela

**Affiliations:** 1 Centre of Marine Sciences, Faculty of Sciences and Technology, University of Algarve, Campus of Gambelas, 8005-139 Faro, Portugal; nkatkam@ualg.pt (K.N.G.); mjrodrigues@ualg.pt (M.J.R.); 2 LEPABE-Laboratory of Engineering of Processes, Environment, Biotechnology and Energy, Department of Chemical Engineering, University of Porto, Rua Dr. Roberto Frias s/n, 4200-465 Porto, Portugal; fmalcata@fe.up.pt; 3 Green Colab-Associação Oceano Verde, Universidade do Algarve, Campus de Gambelas, 8005-139 Faro, Portugal; hugopereira@greencolab.com; 4 BioISI—Biosystems & Integrative Sciences Institute, Faculty of Sciences, University of Lisbon, Campo Grande, C8, 1749-016 Lisbon, Portugal; hmgaspar@fc.ul.pt; 5 MARE—Marine and Environmental Sciences Centre, Polytechnic of Leiria, Edifício CETEMARES, Avenida do Porto de Pesca, 2520-630 Peniche, Portugal

**Keywords:** *Tisochrysis lutea*, loliolide, hepatocellular carcinoma

## Abstract

*Tisochrysis lutea* is a marine haptophyte rich in omega-3 polyunsaturated fatty acids (e.g., docosahexaenoic acid (DHA)) and carotenoids (e.g., fucoxanthin). Because of the nutraceutical applications of these compounds, this microalga is being used in aquaculture to feed oyster and shrimp larvae. In our earlier report, *T. lutea* organic crude extracts exhibited in vitro cytotoxic activity against human hepatocarcinoma (HepG2) cells. However, so far, the compound(s) accountable for the observed bioactivity have not been identified. Therefore, the aim of this study was to isolate and identify the chemical component(s) responsible for the bioactivity observed. Bioassay-guided fractionation through a combination of silica-gel column chromatography, followed by preparative thin layer chromatography (PTLC), led to the isolation of two diastereomers of a monoterpenoid lactone, namely, loliolide (**1**) and *epi*-loliolide (**2**), isolated for the first time in this species. The structural elucidation of both compounds was carried out by GC-MS and 1D (^1^H and ^13^C APT) and 2D (COSY, HMBC, HSQC-ed, and NOESY) NMR analysis. Both compounds significantly reduced the viability of HepG2 cells and were considerably less toxic towards a non-tumoral murine stromal (S17) cell line, although *epi*-loliolide was found to be more active than loliolide.

## 1. Introduction

In recent years, natural products derived from microalgal biomass produced at pilot scale have attracted great attention in drug discovery [1]. Because of their unique biochemical pathways and great adaptability to various environmental conditions such as high salinity, low or high temperatures, high light intensities, and a wide pH range, microalgae can be viable alternatives to terrestrial plants in producing highly valuable natural precursors with potential bioactivity [2]. For instance, *Tisochrysis lutea* (Haptophyta), formerly known as *Isochrysis galbana* T-ISO [3,4], is a marine unicellular flagellated microalga with a golden-brown phenotype, widely used as feed for early larval stages of mollusks, fish, and crustaceans in the aquaculture industry. Interestingly, its potential in food applications and as a source of high-value bioactive compounds has been considered [5]. More recently, it has been described that *T. lutea* is able to accumulate significant levels of *n*-3 polyunsaturated fatty acids (PUFAs) such as docosahexaenoic acid (DHA), and of xanthophylls such as fucoxanthin, which play an important role in the prevention and treatment of human diseases [6]. 

In addition, several isolated compounds have been reported to display a wide spectrum of medicinal properties. For example, crude polysaccharides of *T. lutea* have been evaluated concerning their immunomodulatory properties by the induction of IL-1 in murine macrophages [7]. Moreover, the anti-inflammatory activity of the galactosylglycerides and galactosylceramides has also been assayed [2]. *T. lutea* has exhibited promising therapeutic effects by inducing weight loss and decreasing glucose, triacylglycerol (TAG), and cholesterol levels in diabetic rats [8]. In addition, it contains antioxidants [9], compounds with anti-bacterial activity against multidrug-resistant *Mycobacterium tuberculosis* [10], as well as inhibitors of cyclooxygenase (COX-2) [11] and inhibitors of U937 human leukemic monocyte lymphoma cells proliferation [12]. In a previous study published by our group, the crude extracts of *T. lutea* displayed promising antioxidant properties, inhibition of acetylcholinesterase (AChE), and cytotoxicity against tumor cell lines (HepG2) [13].

Nevertheless, to the best of our knowledge, there are no reports on the isolation of compounds from *T. lutea* with cytotoxic activity against hepatocarcinoma HepG2 cells. Hepatocarcinoma is a prevalent cancer of the liver and occurs particularly in patients with underlying chronic liver disease and cirrhosis. It is the fourth leading cause of cancer mortality and third most common cause of cancer-related death worldwide after lung and gastric cancer [14,15]. Despite the existence of a few drugs for the treatment of liver cancer, patients suffer from hepatotoxicity (including liver failure) and drug resistance, which limit the successful outcome of the treatment in most cases [16]. Consequently, there is an increasing demand for finding new therapeutic drugs for the treatment and/or prevention of hepatocarcinoma [17]. Interestingly, marine algal crude extracts have recently been found to be a vital source of pharmaceutically valuable drugs for the treatment of numerous forms of tumors [18]. Hence, we report here for the first time the isolation and structural elucidation of two stereoisomers of a hydroxylated monoterpene lactone, namely, loliolide (**1**) and *epi*-loliolide (**2**), from *T. lutea*. The evaluation of their cytotoxic effects against human HepG2 cells is also discussed in comparison to those against a non-tumoral cell line (S17 cells).

## 2. Results and Discussion

**Figure 1:**
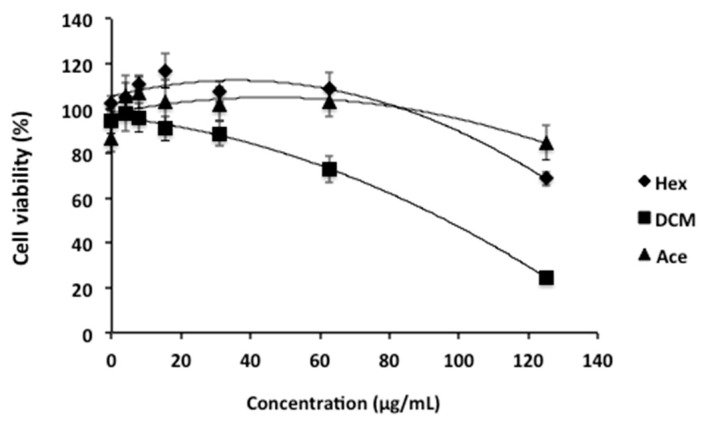
Cytotoxicity of *Tisochrysis lutea* crude extracts of hexane (Hex), dichloromethane (DCM) and acetone (Ace) against HepG2 cell lines.

The extraction was first carried out sequentially using organic solvents with increasing polarity, such as hexane (Hex), dichloromethane (DCM), and acetone (Ace), in order to obtain an enriched bioactive extract. Later, the organic extracts of *T. lutea* were tested for their in vitro cytotoxic activity against HepG2 cells. In our study, dimethyl sulfoxide (DMSO) and the chemotherapeutic drug etoposide were used as negative and positive controls, respectively. Etoposide is a semisynthetic derivative of podophyllotoxin, which is well-known and widely used as an anti-cancer drug [19,20]. It is reported as a topoisomerase II inhibitor, leading to cell-cycle arrest followed by cell death [20]. The MTT colorimetric assay was employed to test the effect of crude extracts of *T. lutea* on mitochondrial metabolic activity (i.e., as an indicator of cell viability). It was found that the DCM extract was the most active compared to the hexane and acetone extracts, decreasing HepG2 cell viability down to 24.7% at a concentration of 125 µg/mL (Figure 1), resulting in an IC_50_ of 85.1 µg/mL (CI_95_% 73.1 to 99.1 µg/mL).

**Figure 2:**
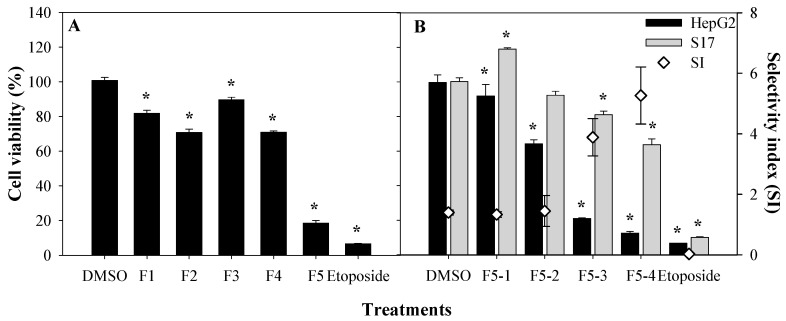
Cell viabilities of fractions F1 to F5 (**A**) against HepG2 cells, and fractions F5–1 to F5–4 towards HepG2 and S17 cells, and the respective selectivity index (**B**). Etoposide was used as positive control. All samples were tested at a concentration of 125 µg/mL. Results are depicted in percentage (%) of cell viability, compared with a negative control (DMSO), tested at a concentration of 0.5% (*v/v*). Values show the mean ± SEM of at least three experiments (*n* = 9). Asterisks (*) indicate significant differences in cell viability between negative control and treated cell lines (*p* < 0.01).

To identify the bioactive molecule(s) responsible for the cytotoxic properties, the active DCM extract was subjected to bioguided fractionation using silica-gel chromatography, which yielded five fractions named as F1 through F5 (Figure 2A). Each fraction was monitored by TLC in order to pool similar fractions. Fraction 5 was the most active towards HepG2 cells, and was further fractionated into fractions F5–1, F5–2, F5–3, and F5–4 by silica-gel column chromatography. These sub-fractions were re-tested at a concentration of 125 µg/mL to evaluate their cytotoxicity against the HepG2 cell line, as well as their selective index (SI) against non-tumoral cell lines sourced from murine bone marrow (S17). The SIs of two subfractions, namely, F5–3 and F5–4, were significantly higher (3.9 ± 0.2 and 5.3 ± 0.5, respectively) than those of F5–1 and F5–2 (SI < 2.0, see Figure 2B) [21,22]. The selectivity of a drug is a highly desirable feature, so that its toxicity is limited to the target (cancer) cells in order to avoid unwanted side effects [23] and also to increase the therapeutic concentration window. Therefore, the selectivity index value indicates the therapeutic potential of a drug; for instance, an SI > 1 means that the drug is less harmful or toxic for non-tumoral cells compared to tumoral cells and is safer for therapeutic applications. An SI > 5 is desired for molecules to be considered as potential drugs with low-toxicity as anti-cancer agents [24]. 

**Figure 3:**
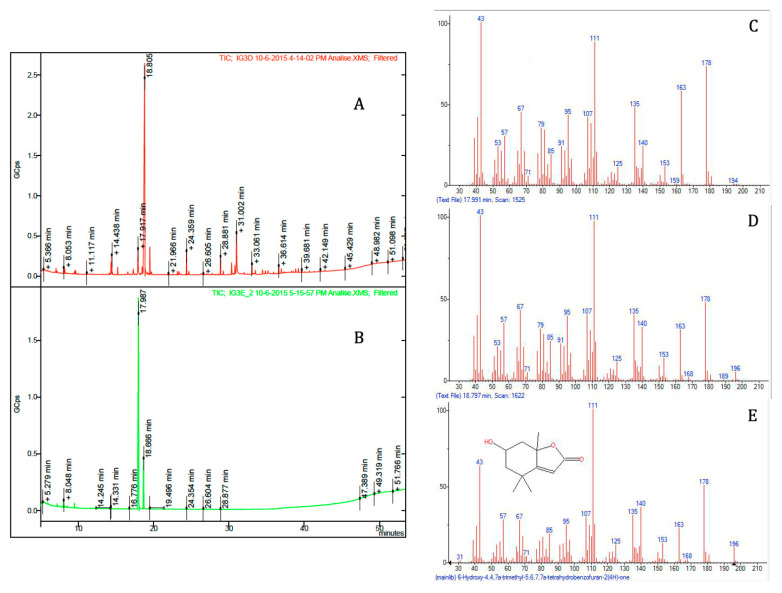
GC-MS chromatograms of sub-fractions F5–3 (**A**) and F5–4 (**B**), and mass spectra of the major peaks of both sub-fractions, at 17.991 min (**C**) and 18.797 min (**D**) and their identification by comparison with the spectrum at the NIST library (**E**).

**Figure 4:**
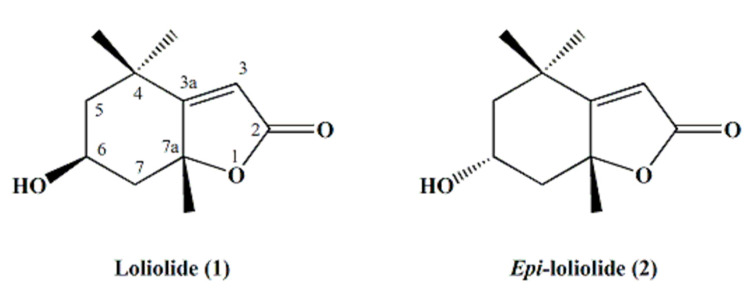
Structures of loliolide (**1**) and *epi*-loliolide (**2**).

The structures of the isolated bioactive compounds present in the subfractions F5–3 and F5–4 were elucidated using spectral analysis, namely, GC-MS, ^1^H and ^13^C NMR. Firstly, subfractions F5–3 and F5–4 were analyzed by GC-MS (Figure 3). Each subfraction contained only one major peak, with retention times of 17.98 min and 18.80 min, respectively (Figure 3A,B). However, the compounds that were found as being the most abundant component of each subfraction had the same molecular ion with *m*/*z* 196 [M^+^], which we tentatively identified as isomers of loliolide by comparing the mass spectral data with the NIST library (Figure 3C–E) and spectrometric data reported elsewhere [25]. The retention factor (Rf) of the two molecules found in subfractions F5–3 and F5–4 were also compared by thin-layer chromatography (TLC), using hexane and ethyl acetate (20:80, *v/v*) as a mobile solvent system. This experiment showed two different spots at 0.43 and 0.34, respectively. To further elucidate the structures of both compounds, 1D (^1^H and ^13^C Attached Proton Test (APT) and 2D (COSY, HMBC, HSQC-ed, and NOESY) NMR spectral analyses were carried out. The chemical shifts and structural assignments (Table 1) were similar to those of loliolide (**1**) and *epi*-loliolide (**2**) (Figure 4) upon comparing with data previously reported in the literature on similar compounds synthesized chemically or found in brown algae [26,27,28,29]. 

These two carotenoid-derived metabolites are monoterpene lactones, also classified as norisoprenoids or apocarotenoids [28,30,31], which have often been isolated from various sources such as plants, algae, and other marine organisms [25,32,33,34]. These types of metabolites are produced from the carotenoids fucoxanthin, zeaxanthin, and violaxanthin upon photo-oxidation or thermal degradation, among other catabolic pathways [33,34]. Carotenoids are well-known dietary supplements used as ingredients in food and cosmeceutical formulations (e.g., as colorants), and perform versatile roles in human health, including neuroprotection and the prevention and/or treatment of macular degeneration, inflammatory and rheumatoid arthritis, cataracts, cancer, diabetes, and coronary artery conditions, among others [35]. Both pigments, fucoxanthin and zeaxanthin, have been reported in *Tisochrysis lutea* [36], which might explain the presence of those monoterpene lactone diastereomers in this marine microalga. Indeed, carotenoids are highly prone to oxidation under different environments, such as light, heat, air, or the presence of transition metals or radicals. These conditions may lead to reactions of isomerization, rearrangement, oxidative cleavage, and/or combinations thereof, resulting in the production of carotenoid catabolites such as norisoprenoids and/or apocarotenoids, including loliolides [37,38]. Interestingly, it has been suggested that the degradation of carotenoids to these smaller molecules might be the reason why these pigments show various biological activities, rather than the original carotenoids themselves. For instance, in a previous report from our laboratory, we presented evidence that another zeaxanthin-derived metabolite (isololiolide), an epimer of loliolide, had significant cytotoxic activity against liver cancer cells (HepG2), while no toxicity was detected for non-tumoral cells derived from MRC-5 (Medical Research Council cell strain 5) of lungs and HFF-1 human fibroblasts [14]. Interestingly, although loliolide (1) isolated from *T. lutea* showed cytotoxicity towards HepG2 and S17 cell lines, this effect was lower than that of *epi*-loliolide (2); this result might be explained by a different configuration around C–6 and/or C–7a (Figure 3), which may account for the diverse bioactive properties of both loliolides [38]. Indeed, isomer-specific bioactivity is often found in nature—for example, the *cis*-isomer of β-carotene has gained more attention due to its higher bioavailability over the corresponding *trans*-isomer [39]. 

From a biological point of view, stereoisomers of carotenoid metabolites, such as loliolide, have been described as having various biological activities, such as potent germination inhibition and ant-repellence; they also showed immunosuppressive and anticholinesterase effects and antioxidant properties, and the ability to prevent H_2_O_2_-induced cell damage [40,41,42]. Loliolide was demonstrated to have antiapoptotic and antiscratching effects in human keratinocytes [43]. In addition, loliolide and isololiolide exhibited strong growth-inhibitory properties on cress and barnyard grass seedlings and anti-melanogenetic activity [30,32,44]. Therefore, the wide spectra of biological properties attributed to loliolide and its isomers [45,46,47,48,49,50] is a strong indication that further research is needed to fully understand the effects of these carotenoid catabolites on either animal, plant, or microbial cells.

## 3. Materials and Methods

### 3.1. Materials

*Tisochrysis lutea* biomass was procured from NECTON S.A. (Faro, Portugal) as a dark green powder material produced by lyophilization. Human hepatocellular carcinoma (HepG2) and murine bone marrow stromal (S17 cell) cell lines were kindly provided by Dr. Vera Marques and Dr. Nuno Santos, Center for Molecular and Structural Biomedicine (CBME), University of Algarve (Faro, Portugal), respectively. Hexane (Hex), methanol, ethyl acetate (EA), dichloromethane (DCM), acetone (Ace), and dimethyl sulfoxide (DMSO) from Fisher Scientific (Loughborough, UK) and 3-(4,5- dimethylthiazol-2-yl)-2,5-diphenyltetrazolium bromide (MTT; Merck, Lisbon, Portugal) were purchased from VWR International (Lisbon, Portugal). TLC-plates ALUGRAM^®^ Xtra Sil G/UV_254_, pre-coated with silica gel 60 (1 mm) and silica gel (70–130 mesh), were purchased from M/s Merck (Lisbon, Portugal). All the other chemicals used were of reagent grade.

### 3.2. Extract Preparation from Microalgae

Lyophilized biomass (50 g) of *Tisochrysis lutea* was dispersed in the chosen solvent and homogenized using a disperser IKA T10B Ultra-Turrax at room temperature. The extractions were made sequentially using 200 mL of hexane, dichloromethane, and acetone at room temperature to obtain crude extracts. The extractions were done in triplicate, filtered through Whatman filter paper n° 4, and the supernatants combined and concentrated under reduced pressure using a rotary evaporator, at 40 °C. All extracts were dissolved in DMSO at a concentration of 50 mg/mL, aliquoted, and stored at 4 °C until use.

### 3.3. Cellular Viability

Cell lines were maintained in culture media RPMI-1640 supplemented with glucose (1000 mg/mL), 10% fetal bovine serum (FBS), L-glutamine (2 mM), streptomycin (50 µg/mL), and penicillin (50 µg/mL). Both HepG2 and S17 cell lines were seeded on 96-well plates, incubated under 5.0% CO_2_ in humidified atmosphere at 37 °C overnight, and later treated with the extracts (hexane, DCM, and acetone) at concentrations ranging from 3.9 to 125 µg/mL for 72 h. Negative controls for cell lines were performed with DMSO at a maximum concentration of 0.5% (*v/v*). The MTT colorimetric assay was carried out to assess their effect on mitochondrial metabolic activity, as an indicator of cell viability [22]. Briefly, two hours before the completion of the incubation period, 20 µL of MTT (5 mg/mL in PBS) were added to each well and further incubated for 2 h at 37 °C. Absorbance was measured at 590 nm using a Multi-Mode Microplate Reader (BioTek Synergy ^TM^ 4, Winooski, VT, USA), and results were calculated as percent of cell viability and as IC_50_ values (µg/mL). Furthermore, the selective index (SI) of the subfractions (F5–1, F5–2, F5–3, and F5–4), and positive control etoposide was evaluated at a concentration of 125 µg/mL. The SI was calculated using the following equation: SI = VNT/VT, where VNT and VT represent the cell viability of non-tumoral cells (S17) and tumoral cells (HepG2), respectively, after exposure to the same concentration of extract or fraction [22].

### 3.4. Bio-Guided Fractionation and Isolation of Anti-Tumoral Compounds 

The DCM crude extract (DCM; 10.8 g) was subjected to a bioguided fractionation using silica-gel (70–130 mesh) column chromatography eluting with mixtures of hexane, ethyl acetate, and methanol of increasing polarity to obtain five fractions as follows: F1 (pure hexane), F2 (Hex:EA, 90:10, *v/v*), F3 (Hex:EA, 70:30 *v/v*), F4 (Hex:EA, 50:50 *v/v*), and F5 (Hex:EA, 70:30 *v/v* and EA:methanol, 80:20 *v/v*). After identification of the active fraction (F5, 2.44 g) this was again subjected to silica-gel column chromatographic fractionation to afford fractions F5–1, F5–2, F5–3, and F5–4, which were eluted using mixtures ranging from pure hexane to pure ethyl acetate, particularly fractions F5–3 and F5–4, which were obtained in a solvent system containing 40–50% hexane in ethyl acetate. The resulting active fractions (F5–3 and F5–4) were further purified by preparative TLC which yielded 29 mg of F5–3 (**1**) and 13 mg of F5–4 (**2**). These pure compounds were stored at 4 °C until further use, such as structural elucidation and biological activities evaluation.

### 3.5. Spectral and Chromatographic Analysis

Pure samples of loliolide (1) and *epi*-loliolide (2) were dissolved in deuterated chloroform (CDCl_3_, Sigma-Aldrich, Switzerland) and analyzed by NMR. ^1^H (400.1 MHz) and ^13^C (100.6 MHz) NMR were recorded on a Bruker Avance spectrometer (Wissembourg, France); chemical shifts were expressed in δ values and referenced to the residual CDCl_3_ peak (δ_H_ = 7.26 ppm and δ_C_ = 77.00 ppm); coupling constants were reported in hertz (Hz). Unequivocal assignments of all proton and carbon signals were achieved by 1D (^1^H, and ^13^C APT) and 2D (COSY, HMBC, HSQC-ed, and NOESY) NMR experiments. The NMR data obtained for both compounds were in accordance with previously reported data [25,26,27,28]. GC-MS analysis was performed using an Agilent 6890N Gas Chromatograph connected to Bruker GC-MS Triple Quad MS System (Model SCION 456-GC; Billerica, MA, USA) at 70 eV (*m/z* 33–1000; source at 230 °C and quadruple at 150 °C) in EI mode with a ZB-5 ms capillary column (30 m × 0.25 mm; 0.25 µm). The column temperature was initially maintained at 60 °C for 1 min, and gradually increased as follows: 60 °C to 120 °C at 30 °C/min, 120 °C to 250 °C at 4 °C/min, then 250 °C to 270 °C at 20 °C/min, and finally 270 °C to 300 °C at 2.5 °C/min, where it remained for 5 min. The carrier gas was helium at a flow rate of 1.0 mL/min, the inlet temperature was maintained at 300 °C and split-less mode was used.

## 4. Conclusions

Our results indicate that crude extracts and/or biomass of *T. lutea* can be a source of compounds for the prevention and treatment of human hepatocarcinoma. Two cytotoxic and selective compounds were isolated, loliolide (**1**) and *epi*-loliolide (**2**), which are likely to be degradation products of fucoxanthin and/or zeaxanthin. Therefore, comparative biological studies of pure carotenoids (e.g., fucoxanthin and zeaxanthin) and their respective catabolites are warranted in order to find a structure–activity relationship (SAR) of loliolide and its stereoisomers. A possible research avenue would include the modification of the hydroxyl group occurring in loliolides in order to generate various analogues and determine their bioactivities, which could further improve the usefulness of these lactones.

## Figures and Tables

**Table 1:** Chemical shifts (^1^H and ^13^C NMR) of loliolide and *epi*-loliolide.

Position	Loliolide (1)		*Epi*-loliolide (2)	
	*δ*C	*δ*H, m, *J* (Hz)	*δ*C	*δ*H, m, *J* (Hz)
2	171.49	-	171.55	-
3	112.89	5.69 s, 1H	113.24	5.71 s, 1H
3a	182.68	-	180.75	-
4	35.91	-	35.03	-
5	47.26	1.53 dd, 14.5, 3.3, 1H, α-H*_ax_*	49.7	1.33 t, 12.8, 1H, β-H*_ax_*
		1.97 brd, 14.6, 1H, β-H*_eq_*		2.04 brd, 12.8, 1H, α-H*_eq_*
6	66.81	4.33 m, 1H, α-H*_eq_*	65.03	4.13 tt, 11.5, 4.1, 1H, α-H*_ax_*
7	45.56	1.79 m, 1H, α-H*_ax_*	47.25	1.51 t, 11.9, 1H, β-H*_ax_*
		2.45 brd, 14.1, 1H, β-H*_eq_*		2.54 brd, 11.8, 1H, α-H*_eq_*
7a	86.67	-	86.45	-
4α-Me	30.63	1.27 s, 3H, Me*_eq_*	25.04	1.26 s, 3H, Me*_ax_*
4β-Me	26.45	1.46 s, 3H, Me*_ax_*	29.89	1.31 s, 3H, Me*_eq_*
7a-Me	26.96	1.78 s, 3H, β-Me*_ax_*	25.55	1.58 s, 3H, α-Me*_ax_*
